# The Protective Role of Vitamin E against Oxidative Stress and Immunosuppression Induced by Non-Esterified Fatty Acids in Bovine Peripheral Blood Leukocytes

**DOI:** 10.3390/ani14071079

**Published:** 2024-04-02

**Authors:** Cheng-Yan Li, Wei-Chen Lin, Tossapol Moonmanee, Jacky Peng-Wen Chan, Chien-Kai Wang

**Affiliations:** 1 Department of Animal Science, National Chung Hsing University, Taichung 402202, Taiwan; beagleis0912@hotmail.com (C.-Y.L.); weichenlin728377@gmail.com (W.-C.L.); 2 The iEGG and Animal Biotechnology Center, Rong Hsing Research Center for Translational Medicine, National Chung Hsing University, Taichung 402202, Taiwan; 3 Department of Animal and Aquatic Sciences, Faculty of Agriculture, Chiang Mai University, Chiang Mai 50200, Thailand; tossapol.m@cmu.ac.th; 4 Functional Feed Innovation Center, Faculty of Agriculture, Chiang Mai University, Chiang Mai 50200, Thailand; 5 Department of Veterinary Medicine, National Chung Hsing University, Taichung 402202, Taiwan; pwchan@dragon.nchu.edu.tw

**Keywords:** non-esterified fatty acids (NEFA), negative energy balance (NEB), vitamin E, oxidative stress, peripheral blood leukocytes, pro-inflammatory, transition period

## Abstract

**Simple Summary:**

During the transition period, dairy cows mobilize fat reserves to produce non-esterified fatty acids (NEFAs) as an additional energy source to meet the energy demands for fetal growth, calving, and lactation. However, high levels of serum NEFAs result in increased oxidative stress and disease incidence, and impairment of the immune function of leukocytes in cows. Vitamin E is a critical fat-soluble antioxidant that reduces the impact of oxidative stress on immune cells and enhances the functioning of immune cells during the transition period. In this in vitro study, peripheral blood leukocytes (PBLs) were isolated from dry cows to investigate the effects of high NEFA levels on bovine peripheral leukocytes and to explore the protective role of vitamin E through pre-treatment. The findings indicate that high levels of NEFA induce oxidative stress in PBLs and alterations in cytokine expression, while pre-treatment with vitamin E partially mitigates NEFA effects on bovine PBLs. This may suggest that a serious negative energy balance leads to oxidative stress on immune cells and induces changes in inflammation-related cytokines during cows’ transition period. Supplementation with vitamin E could diminish some of the impacts caused by the negative energy balance on immune cells.

**Abstract:**

High levels of non-esterified fatty acids (NEFAs) during the transition period lead to increased oxidative stress and immunosuppression in cows. Feeding them a vitamin-E-supplemented diet reduces reactive oxygen species (ROS) levels in the blood and diminishes immunosuppression in the transition period. However, whether the restoration of immune cell function occurs through the direct action of vitamin E in cells is still a topic that requires further discussion. Therefore, in this experiment, we aimed to investigate the effect of NEFAs on peripheral blood leukocytes (PBLs) and whether vitamin E mitigates the impact of NEFAs. We employed three groups: (1) blank, (2) NEFA only, and (3) pre-culturing with vitamin E before NEFA treatment (VENEFA). In peripheral blood mononuclear cells (PBMCs), there were no differences in vitamin E content among the three groups. However, in the vitamin E pre-treatment group, the vitamin E levels of polymorphonuclear neutrophils (PMNs) were significantly higher than those in the other two groups. NEFA levels increased malondialdehyde (MDA) levels in PBMCs, but pre-treatment with vitamin E reduced accumulation of MDA levels. Regarding the expression of proinflammatory genes, NEFAs increased the expression of interleukin-1β in PBMCs and colony-stimulating factor 2 in PMNs. Vitamin E pre-treatment restored the increase in interleukin-1β levels caused by NEFAs in PBMCs. None of the groups affected the phagocytosis of PMNs. Few studies have confirmed that NEFAs cause oxidative stress in bovine PBLs. In summary, this study found that NEFAs induce oxidative stress in PBLs and alter the expression of inflammation-related genes; meanwhile, vitamin E can reduce some of the effects caused by NEFAs. This result may suggest that vitamin E can assist bovine PBLs in resisting the immune suppression caused by an NEB during the transition period.

## 1. Introduction

During the transition period, increased energy demands in cows lead to a negative energy balance (NEB), a significant reduction in dry matter intake, and a delayed recovery of feed intake, all of which lead to a more severe NEB at this stage. In order to satisfy these energy demands, cows increase fat mobilization to produce non-esterified fatty acids (NEFAs) and ketone bodies, which indicate the degree of adaptation to an NEB [1] but also increase reactive oxygen species (ROS) generation and oxidative stress levels during the transition period [2]. Higher serum NEFA concentrations increase the risk of postpartum diseases [3] and are considered a vital health regulatory factor during the transition period, especially for immune cell function. Abnormal peripheral blood leukocyte counts have been observed in various diseases, such as subclinical ketosis [4] and retained placenta [5]. Moreover, the immune function of peripheral leukocytes is suppressed during the transition period, involving issues such as decreased cell proliferation, increased secretion of proinflammatory cytokines, and reduced phagocytic activity [6]. Culturing with high concentrations of NEFA results in impaired cell functions for bovine neutrophils [7], induces the expression of pro-inflammatory cytokines in peripheral leukocytes, and intensifies the inflammatory response induced by lipopolysaccharides [8]. Hence, higher NEFA levels may disrupt the immune cell functions of bovine peripheral leukocytes and increase the health risks posed to dairy cows during the transition period.

The colony-stimulating factor (CSF) family, most well known for its hematopoietic functions, has also been proven to regulate various immune cell functions. For example, with CSF-2 (known as granulocyte-macrophage colony-stimulating factor, GM-CSF), knock-out mice show reduced neutrophil function [9], and CSF-1 (known as macrophage colony-stimulating factor, M-CSF) has been observed to increase the Th1/Th2 ratio in ovarian cancer patients [10]. In the study of dairy cows, the administration of CSF-3 (known as granulocyte colony-stimulating factor, G-CSF) from 14 days prepartum to 10 days postpartum has been shown to reduce immunosuppression in the transition period [11,12]. Injecting CSF-3 also increases white blood cell counts during the transition period; even in an NEB status, it is suggested to enhance neutrophils’ myeloperoxidase release [13]. CSFs have been confirmed to be involved in the expression of other proinflammatory cytokines [14]. The above explanation clarifies that the CSF family is an important immunomodulatory factor, but there has been limited discussion regarding its expression in bovine peripheral blood leukocytes.

Vitamin E plays a crucial role as a fat-soluble antioxidant and has been shown to optimize immune cell function. During the transition period in dairy cows, there are significant changes in vitamin E levels and oxidative stress indicators in the blood. Plasma alpha-tocopherol concentrations tend to be lowest around calving and gradually increase postpartum [15], while oxidative stress increases around calving [16]. Supplementing vitamin E reduces postpartum plasma MDA concentrations [16] and mitigates immunosuppression during the transition period [17,18]. The trace element selenium (Se) can reduce the accumulation of hydroperoxides through selenoproteins, such as glutathione peroxidase and thioredoxin reductases [19]. Therefore, during the transition period, supplementation with Se yeast can elevate the concentration of vitamin E in the bloodstream [20]. Nevertheless, it is important to note that the relationship between oxidative indicators and vitamin E levels can vary in different tissues [21]. Additionally, there is a low correlation between vitamin E levels in erythrocytes and plasma during the transition period [22]. Hence, it is essential to approach the evaluation of oxidative stress using circulating vitamin E levels with caution, considering the potential variations across different tissues.

Many in vivo studies have demonstrated that administering vitamin E through feeding or injection can improve the function of PBLs in cows. However, it remains unclear whether this improvement occurs directly through increased cellular vitamin E levels, particularly in cases of high NEFA levels (a severe NEB). This study aims to investigate the effects of high levels of NEFAs and the protective influence of vitamin E on bovine PBLs. First, we assess the influence of NEFAs and vitamin E in the culture environment on the vitamin E content in bovine PBLs. Then, we further discuss their effects on cellular oxidative stress and the expression of inflammation-related genes to clarify NEFAs’ impacts on bovine PBLs and whether pre-treatment with vitamin E mitigates the impacts induced by high levels of NEFAs. These findings may help to control the health risks in severe NEB cows during the transition period.

## 2. Materials and Methods

### 2.1. Ethical Approval

The animal research procedures in this experiment were approved and supervised by the Institutional Animal Care and Use Committee of the National University (Certificate Number: IACUC-109-150).

### 2.2. Animals and Cell Sampling

Blood samples and PBLs were collected from ten dry cows with parity ranging from 2 to 3 (average age = 5.37 ± 0.39), sourced from the National Chung Hsing University farm. Blood samples amounting to 100 mL were collected from the jugular vein of each cow around 4 to 8 weeks before the expected calving date for PBL isolation. To understand the impact of NEFAs on the vitamin E content in peripheral blood leukocytes and further explore whether a culturing environment with sufficient vitamin E counteracts the effects of NEFAs on cellular vitamin E, we utilized peripheral blood leukocytes from dry cows to avoid the influence of endogenous NEFAs. Blood samples were transferred into ethylenediaminetetraacetic acid- (EDTA-) coated tubes (BD Vacutainer EDTA tube, Becton, Dickinson, and Company, Franklin Lakes, NJ, USA), and then cell isolation was performed. All animals were kept in free stalls and provided ad libitum access to hay (50% Bermuda hay and 50% Pangola hay). Each animal was fed 1.5 kg of concentrate (including cracked corn, wheat bran, and soybean meal with 20.4% crude protein, 20.3% neutral detergent fiber, 120 IU/kg vitamin E, and 0.95 mg/kg selenium) twice daily at 7:00 a.m. and 3:00 p.m. This procedure complies with the nutritional requirements for dry dairy cows recommended by the US National Research Council in the seventh revised edition of its guidelines [23].

### 2.3. Isolation of Bovine PBMCs and PMNs

Blood samples were diluted with equal volumes of 1× Hank’s balanced salt solution (HBSS; Gibco-BRL Life Technologies, Waltham, MA, USA). Subsequently, 25 mL of each diluted blood sample was slowly added to a 50 mL centrifuge tube containing 15 mL of Lymphoprep™ 1.077 (Axis-Shield PoC AS, Oslo, Norway). The solution was centrifuged at room temperature at 700× *g* for 30 min. PBMC and PMN suspensions were collected from the upper white layer and the bottom layer, respectively. We added a 5× volume of erythrocyte lysis buffer (1 L of deionized distilled water with 8.26 g of ammonium chloride, 1 g of potassium bicarbonate, and 0.037 g of EDTA) [24] to the cell suspension and incubated it for 10 min to allow for erythrocyte lysis. Then, an equal volume of HBSS was added to terminate the action of the lysis buffer and wash the cell pellets. We centrifuged the solution at 400× *g* for 10 min and removed the supernatant. We then repeated the above steps twice to thoroughly eliminate residual erythrocytes. The collected PBMCs and PMN cell pellets were suspended in an RPMI medium containing 5% fetal bovine serum and 1% penicillin–streptomycin solution and set aside for an in vitro culture.

### 2.4. NEFE, Vitamin E, and Medium Preparation

The primary plasma NEFAs in cows during the transition period are stearic acid (C18:0), palmitic acid (C16:0), and oleic acid (cis-9 C18:1) [25]. This study’s NEFA preparation method was based on that presented in [26], with modifications. We prepared a stock solution containing 34.35 mM of oleic acid (OA, O1383, Sigma-Aldrich, St. Louis, MO, USA), 25.2 mM of palmitic acid (PA, P0500, Sigma-Aldrich, St. Louis, MO, USA), and 11.4 mM of stearic acid (SA, S4751, Sigma-Aldrich, St. Louis, MO, USA). After being filtered through a 0.45 μm PVDF filter (Merck Millipore, Tullagreen, Carrigtwohill, Ireland), the solution was ready for subsequent use in culturing. The method used to prepare the vitamin E stock solution was based on [27]. Using absolute ethanol, we prepared 712 mg/mL of alpha-tocopherol. To enhance cellular uptake, the vitamin E solution was added to FBS, resulting in a concentration of 84 mg/mL, and incubated at 37 °C for one hour. We added the vitamin E and FBS mixture to an RPMI medium to create a 300 μg/mL vitamin E stock solution. The basal culture medium consisted of RPMI-1640 (Gibco, Hercules, CA, USA) supplemented with 10% heat-inactivated FBS (Corning Inc., New York, NY, USA), 2% fatty-acid-free bovine serum albumin (A8806, Sigma-Aldrich, St. Louis, MO, USA), 2 mM L-glutamine (G8540, Sigma-Aldrich, St. Louis, MO, USA), and 1% penicillin–streptomycin (Biological Industries, Beit Haemek, Israel). It was used for subsequent stock dilution and cell culturing.

### 2.5. Cell Culture

**Figure 1:**
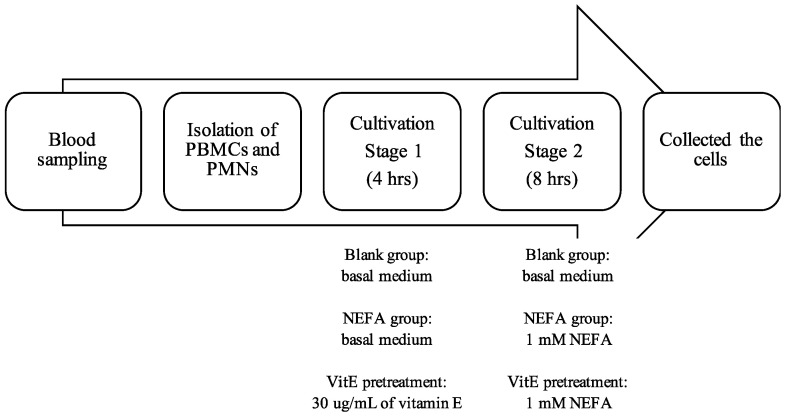
Culture process diagram. Abbreviations: NEFA, non-esterified fatty acids; VitE, vitamin E; PBMCs: peripheral blood mononuclear cells; PMNs: polymorphonuclear neutrophils.

The experiment was divided into three groups: (1) blank, (2) NEFA, and (3) vitamin E pre-treatment (VENEFA). We cultured 10^8^ cells in each well of a 6-well plate (Falcon^®^ 6-well clear flat-bottom not-treated cell multiwell culture plate, 351146, Corning Inc., New York, NY, USA). The first stage was four hours long; herein, the blank and NEFA groups were cultured using a base culture medium. The vitamin E pre-treatment group was cultured using 30 μg/mL of vitamin E medium. After that, we collected the cell suspension and centrifuged it at 400× *g* for 10 min. After removing the supernatant, the cells and culture plate were washed with HBSS. The second stage lasted eight hours; herein, the blank group was cultured using a basal culture medium. The NEFA and vitamin E pre-treatment groups were cultured using 1 mM of NEFA. During the transition period, an increase in NEFA levels is associated with an elevated risk of postpartum diseases [28]. In healthy postpartum cows, plasma NEFA levels are approximately 0.4 mmol/L, while those in cows with ketosis (severe NEB) are as high as 1.18 mmol/L. The median concentration of NEFAs in postpartum cows is 1.03 mmol/L [29]. Therefore, in this study, a concentration of 1 mmol/L NEFA was used to culture immune cells, simulating a typical NEB scenario. After culturing, adherent cells were removed from the dish using a cell scraper (Spatula cell lifter, Trasadingen, Switzerland), and the cell suspension was collected. We centrifuged the cell suspension at 400× *g* for 10 min, removed the supernatant, washed the cells with HBSS, and set the resulting product aside for subsequent testing. The experimental procedure is shown in Figure 1.

### 2.6. Vitamin E Detection

The cellular vitamin E concentration was determined using an Elabscience Vitamin E Colorimetric Assay Kit (E-BC-K033-S, Elabscience, Richmond, BC, CA). This assay involves the reduction of Fe^3+^ to Fe^2+^ by vitamin E in the presence of ferrion. Fe^2+^ then reacts with phenanthroline, resulting in a pink solution, and the absorbance is measured at 533 nm. This enables the quantification of vitamin E content using a standard curve. To perform this assay, a homogenization reagent (reagent 4) was added to the pellets, followed by vortexing at 2500 rpm for 10 min. The mixture was then centrifuged, and the supernatant was collected. N-heptane and absolute alcohol were added to the supernatant, and the mixture was vortexed before being centrifuged at 4000 rpm for 10 min. This process produced an n-heptane vitamin E extraction solution, which was used for subsequent colorimetric reactions. A chromogenic agent (Reagent 1) and a ferrum reagent (Reagent 2) were added to the n-heptane vitamin E extraction solution, and the mixture was allowed to incubate for five minutes. Subsequently, a stop solution (Reagent 3) and absolute alcohol were added. The absorbance was measured at 533 nm, and the concentration of vitamin E was calculated accordingly.

### 2.7. SOD Activity Detection

The Superoxide Dismutase Assay Kit (Cayman Chemical, Ann Arbor, MI, USA) was utilized to determine superoxide dismutase enzyme activity. This assay involves the generation of superoxide using xanthine oxidase and hypoxanthine. Subsequently, tetrazolium salt is reduced to formazan dye, and the superoxide dismutase (SOD) activity is estimated based on absorbance values. In summary, a diluted radical detector and xanthine oxidase were added to the cell lysates, and the mixture was placed on a shaker shielded from light for 30 min. Following incubation, the absorbance of the mixture was measured at 450 nm. The obtained OD value was then fitted to the standard curve to calculate SOD activity.

### 2.8. TABRS Detection

Malondialdehyde (MDA) is a lipid peroxidation product commonly used to assess cellular oxidative stress. The Thiobarbituric Acid Reactive Substances (TBARS) assay kit (10009055, Cayman Chemical, Ann Arbor, MI, USA) was used to determine cellular MDA levels in this study. SDS solution and color reagent were added to vials containing cell lysate or a standard to perform this assay. The mixture was then incubated in a water bath at 100 °C for 1 h. Subsequently, the vials were placed on ice for 10 min to terminate the reaction. Afterward, the samples were centrifuged at 1600× *g* for 10 min to collect the supernatant, which was allowed to stand at room temperature for 30 min. Finally, 150 µL of the supernatant was transferred into a 96-well plate, and the absorbance was immediately measured at 530 nm.

### 2.9. Determination of Cytokine mRNA Expression

Total RNA extraction was carried out using TRIzol reagent (Invitrogen, Carlsbad, CA, USA). The quality of the extracted total RNA was assessed by measuring the absorbance ratio at 260/280 and via gel electrophoresis. Subsequently, the SuperScript IV Reverse Transcriptase kit (Invitrogen, Carlsbad, CA, USA) was employed to perform the reverse transcription of 2 µg of total RNA, generating cDNA. The synthesized cDNA was then subjected to a quantitative polymerase chain reaction (qPCR) using iTaqTM Universal SYBR^®^ Green Supermix (Bio-Rad, Hercules, CA, USA) to evaluate the expression differences of the target gene following various treatments. The primer sequences utilized are listed in Table 1. A ΔCt calculation was conducted, using GAPDH, which has been validated as a reliable reference gene in cattle, known as the housekeeping gene [30]. By subtracting the ΔCt of the blank group from the obtained ΔCt, the ΔΔCt was obtained. The gene expression difference was determined using the following formula: 2^−(ΔΔCT)^.

### 2.10. Phagocytosis

Using the CytoSelect™ 96-Well Phagocytosis Assay (Cell Biolabs Inc., San Diego, CA, USA), we assessed PMN phagocytic ability by culturing 2 × 10^5^ PMNs in each well of a 96-well plate. First, in the vitamin E pre-treatment group, the culture medium contained 30 μg/mL vitamin E, while the blank and NEFA groups were cultured using a basal culture medium, all for 4 h. Next, the 96-well plate was centrifuged at 250× *g* for 10 min. After removing the supernatant, a basal culture medium was added to the blank group. For the NEFA and vitamin E pre-treatment groups, a culture medium containing 1 mM of NEFA was added. All treatments were then incubated for 6 h at 37 °C with 5% CO_2_. Afterward, E. coli suspension was added to the plates and cultured for 6 h. Upon completion of the culture, we removed the supernatant and added fixation solution, allowing it to sit for five minutes. The plates were then washed twice with DPBS, and blocking reagent was added; then, shaking was applied for 30 min. After removing the supernatant, the plates were washed twice with DPBS. A permeabilization solution was added and left to sit for five minutes, followed by two washes with DPBS. Subsequently, a substrate was added and left to stand for 30 min. Finally, a stop solution was added, and the absorbance was measured at 450 nm.

### 2.11. Statistical Analysis

The quantified data in this experiment are presented as the mean ± standard error (SE). Differences among various treatment groups regarding cellular vitamin E content, cytokine expression, oxidative stress markers, and phagocytic capacity were analyzed for significance differences using one-way analysis of variance (ANOVA) followed by LSD post hoc analysis. Statistical significance was analyzed using IBM SPSS Statistics 29 (IBM, New York, NY, USA) and was considered significant when *p* < 0.05.

## 3. Results

### 3.1. Cellular Vitamin E Content

**Figure 2:**
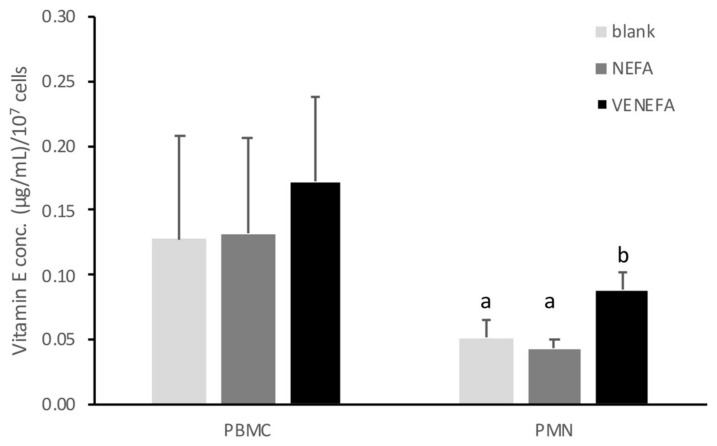
Effect of NEFA and vitamin E pre-treatment combined with NEFA on PBMC (*n* =5) and PMN (*n* = 7) cellular vitamin E concentration. Data are presented as means ± standard error. The different letters indicate significant differences among treatments. (*p* < 0.05). Blank: basal culture medium treatment (4 h) + basal culture medium treatment (8 h). NEFA: basal culture medium treatment (4 h) + 1 mM NEFA treatment (8 h). VENEFA: 30 μg/mL vitamin E treatment (4 h) + 1 mM NEFA treatment (8 h).

Treatment with 1 mM of NEFAs had no effect on the levels of cellular vitamin E in both PBMCs and PMNs compared with the blank group (Figure 2) (blank: NEFA group; PBMCs, 0.13 ± 0.08 vs. 0.13 ± 0.07; PMNs, 0.05 ± 0.01 vs. 0.04 ± 0.01 µg/mL). Pre-treatment with 30 μg/mL of vitamin E for 4 h before NEFA treatment (vitamin E pre-treatment group) significantly increased cellular vitamin E levels in PMNs in comparison with the other two groups (0.05 ± 0.01, 0.04 ± 0.01 vs. 0.09 ± 0.01 µg/mL). In PBMCs, pre-treatment with vitamin E slightly increased vitamin E content (0.17 ± 0.06 vs. 0.13 ± 0.08, 0.13 ± 0.07 μg/mL). Nevertheless, the difference was not statistically significant compared to the other two groups due to high variability (Figure 2).

### 3.2. Oxidative Stress (SOD Activity and TABRS)

Increased oxidative stress during the transition period is considered to be associated with elevated NEFA levels. In this experiment, we aimed to understand the impact of NEFAs on oxidative stress in PBLs and investigate whether a culture environment with higher vitamin E can assist cells in resisting the effects caused by NEFAs. NEFA treatment did not alter SOD activity in PBMCs and PMNs. However, NEFA treatment leads to higher levels of TBARS in PBMCs compared to the blank group (3.86 ± 0.34 vs. 3.01 ± 0.25 µM), while PMN levels did not significantly increase (Table 2). The SOD activity with vitamin E pre-treatment in PBMCs and PMNs was not different from that in the other two groups. Vitamin E pre-treatment reduced the levels of TBARS in PBMCs compared to the NEFA group (3.13 ± 0.24 vs. 3.86 ± 0.34 µM) and showed no difference compared to the blank group (3.13 ± 0.24 vs. 3.01 ± 0.25 µM) (Table 2).

### 3.3. Inflammation-Related Cytokine Gene Expression and Phagocytosis

NEFA treatment significantly increased the expression of *IL-1β* in PBMCs compared to the blank group (1.7 ± 0.08 vs. 1 ± 0.02 folds). However, NEFA treatment did not cause a difference in *IL-1 β*, *IL-6*, and *IL-10* expression in PMNs. The expression of the colony-stimulating factor (CSF) family in bovine PBLs is rarely discussed, especially in the NEB condition. Interestingly, NEFA treatment significantly increased the expression of CSF2 in PMNs compared to the blank group (1.82 ± 0.28 vs. 1 ± 0.06 folds), while PBMCs only showed a trend of a difference in CSF3 expression (Table 3). We further compared the effects of vitamin E pre-treatment. Vitamin E pre-treatment lowered *IL-1* expression in PBMCs compared to the NEFA group (1.35 ± 0.17 vs. 1.7 ± 0.08 folds) and CSF1 expression in PMNs compared to the blank group (0.61 ± 0.09 vs. 1 ± 0.03) (Table 3).

Phagocytosis is one of the crucial functions of neutrophils, enabling them to eliminate pathogens swiftly. There was no significant difference in phagocytic function among the three treatments (blank, NEFA, VENEFA; 0.35 ± 0.03, 0.43 ± 0.05, 0.34 ± 0.03 optical density (450 nm)). Although NEFA treatment showed slightly increased phagocytic ability compared to the blank group, the group variability was high and no significant difference was observed.

## 4. Discussion

During the transition period in cows, energy requirements increase and dry matter intake gradually decreases prepartum [31], resulting in a negative energy balance [32]. Therefore, there is an increase in fat mobilization and a decrease in backfat thickness [33], leading to the hydrolysis of triglycerides to produce NEFAs and glycerol. This increases NEFA concentrations in the blood and enhances the efficiency of NEFA absorption by the liver. Approximately 20% of NEFAs undergo hepatic circulation and are absorbed by the liver [34]. However, ruminants have a relatively poor capacity to synthesize and secrete very-low-density lipoprotein (VLDL) from the liver, accumulating TG in the liver [35,36]. Therefore, severe NEB and NEFA generation may lead to a fatty liver, further impacting cattle metabolism. Ketotic cows exhibit higher plasma concentrations of NEFA and BHBA, along with elevated MDA levels and reduced SOD expression [37]. Moreover, NEFAs are positively correlated with MDA levels and negatively correlated with SOD [38], indicating a strong correlation between blood lipid metabolism markers and oxidative stress. Calf primary hepatocytes treated with NEFA showed an increase in ROS and a decrease in catalase, and these results revealed a dose-dependent effect [39]. In bovine mammary epithelial cells, treatment with 0.9 mM of NEFA increased MDA and decreased SOD activity [40]. However, little research directly demonstrates the effect of NEFA on oxidative stress indicators in bovine PBLs. In the present study, a 1 mM NEFA treatment resulted in a significant increase in MDA accumulation in PBMCs, with no significant increase observed in PMNs. However, SOD activity remained unchanged in both cell types. A study on cattle compared blood oxidative stress levels between late pregnancy and early lactation periods. Cows in the early postpartum stage showed higher MDA levels. However, the activities of SOD and catalase (CAT) remained unchanged, while the glutathione (GSH) levels decreased significantly [41], suggesting that various antioxidants regulate MDA levels together. Such findings may explain the unchanged superoxide dismutase activity observed in this in vitro experiment. Additionally, NEFA only induces oxidative stress in PBMCs, suggesting different tolerances for NEFA-induced oxidative stress between the two cell types. This phenomenon may be attributed to the different proportions of fatty acids on the cell membrane. Increasing evidence suggests that increased double bonds in PUFAs in cellular lipids mediate oxidative stress induced by free radicals [42,43]. Lymphocytes have a relatively high content of free fatty acids in their cell membranes, making them more susceptible to peroxidative damage [44]. During the cultivation process, the oxidation of palmitic acid in lymphocytes is greater than that in neutrophils [44]. These results indicate that PBMCs are more susceptible to the oxidative stress induced by NEFA.

Vitamin E is a critical lipid-soluble antioxidant; this is particularly true for alpha-tocopherol, the primary antioxidant localized in cell membranes. The rate at which vitamin E binds with peroxyl radicals is faster than its binding with polyunsaturated fatty acids (PUFAs). It reduces the impact of peroxyl radicals (ROO•) on PUFAs in cell membranes, thereby terminating radical chain reactions, reducing the effect of oxidative stress on cells. In cattle studies, the feeding or injection of vitamin E to enhance immune cell function has been extensively discussed [45,46]. In a study examining PMN functionality, the culture of PMNs with high concentrations of NEFA did not significantly impact phagocytic activity [7]. This finding is the same as the result of our study. Furthermore, supplementing vitamin E during the transition period did not increase oxidative burst in PMNs [47]. However, another study reported that vitamin E supplementation during the transition period did enhance PMN phagocytic activity [48]. It is important to note that this study also included an additional 20% concentrate feed in the vitamin E supplementation group, suggesting that the observed effects may not solely be attributed to vitamin E. These findings imply that vitamin E supplementation alone may not fully address the functional deficiencies in PMNs during the transition period. Co-treatment with vitamin E (0.5 ng–50 μg) and concanavalin A in lymphocytes has shown a dose-dependent enhancement of proliferation [49]. To the best of our knowledge, there is still limited research on the direct impact of vitamin E supplementation on the vitamin E content and oxidative stress in bovine PBLs, particularly in situations involving an NEB. Moreover, there is a low correlation between blood vitamin E content and blood cell vitamin E in cows [22]. Therefore, it is worth exploring the impact of PBLs on vitamin E absorption. In this study, following culturing with NEFA and pre-treatment with vitamin E, we observed changes in vitamin E content in bovine PBLs. When cultured with 1 mM of NEFAs, no changes were observed in the cellular vitamin E content of PBMCs and PMNs. However, in the vitamin E pre-cultivation group, it was found that PMNs could uptake vitamin E from the culture medium. Even after subsequent NEFA cultivation, the vitamin E content remained significantly higher than that of the control group. Regarding oxidative stress, NEFAs increased MDA in PBMCs, which could be reduced by pre-cultivation with vitamin E. The SOD activity of PMNs showed a slight increase. Nevertheless, due to significant variability, no significant differences were observed between the control group and the NEFA group. In previous studies, feeding cattle 2500 IU/d of vitamin E during the two weeks before and after calving significantly increased the vitamin E content in both plasma and neutrophils [50]. This result is consistent with the results of this study’s in vitro experiment. However, this study did not examine PBMCs or explore the oxidative stress situation in PBLs. It also provided no evidence of lipid metabolism status in cows. This study offers additional research information that allows a more detailed discussion of how vitamin E and NEFA affect different types of PBLs.

Fatty acids are important immune modulators in various animal models [47]. NEFA regulates gene expression in cells directly or indirectly through multiple receptors, including toll-like receptors, peroxisome proliferator-activated receptors, and FFA receptors. However, the effects of saturated and unsaturated fatty acids on oxidative stress and cellular function vary. Fatty acid desaturase is an enzyme that introduces double bonds into fatty acyl chains, thereby converting saturated fatty acids into unsaturated fatty acids. In a mouse model of non-alcoholic fatty liver disease, it was observed that delta-5 desaturase (D5D) and D9-16D are inversely associated with TBARS and ROS levels [51]. Stearoyl-CoA desaturase-1 (SCD-1) is an enzyme involved in converting saturated fatty acids into monounsaturated fatty acids. Human studies suggest that SCD1 plays a role in regulating immune cell differentiation [52]. Furthermore, the activity of SCD1 in bovine cumulus cells has been found to protect oocytes from the adverse effects of saturated fatty acids. These findings indicate the involvement of fatty acid desaturase in modulating fatty acid toxicity and oxidative stress. However, more relevant studies focusing on bovine PBLs are currently needed. In bovine hepatocytes and endometrial cells, exposure to NEFA reduces intracellular antioxidant levels (such as glutathione, catalase, and glutathione peroxidase) while increasing NFκB expression and the expression of pro-inflammatory cytokines. However, the effects caused by NEFA can be alleviated by antioxidants [39,53]. In this study, culturing with NEFA significantly increased *IL1B* expression in PBMCs. There was also a trend of increased *IL10* and CSF3 expression in PBMCs. The expression of *IL1B* in PBMCs could be reduced through vitamin E pre-treatment, indicating that fatty-acid-induced inflammatory expression may be mediated through oxidative stress. The CSF family stimulates the proliferation and differentiation of immune cells and is also a common pro-inflammatory factor. Human type 2 diabetes leads to an increase in NEFA levels in the blood. Stimulating human macrophages with linoleic acid will increase CSF2 expression and demonstrate a dose-dependent effect [54]. This study found that NEFA significantly increases the expression of CSF2 in PMNs. CSF2 may promote the production of reactive oxygen species (ROS) by immune cells and increase the expression of TLR2/4 [55,56], thereby exacerbating the inflammatory response. Culturing with vitamin C reduces the ROS generation caused by CSF1 and decreases the activation of inflammatory pathways [57]. In this study, vitamin E similarly reduced the expression of CSF1 in PMNs. The above results demonstrate that NEFA mediates the expression of inflammation through ROS, while antioxidants reduce the inflammation caused by NEFA, findings that are consistent with those reported in this study. In this study, we further found that PBMCs and PMNs exhibit different levels of resistance to the oxidative effects induced by NEFA, and they also differ in their efficiency of absorbing vitamin E. Therefore, employing multiple antioxidant strategies may be more effective in regulating the inflammatory expression of both peripheral blood leukocytes and enhancing immune function during the transition period. This approach warrants further investigation.

This study has some limitations. Various factors alter the composition of free fatty acids and the concentration of NEFAs, such as dietary ingredients and the metabolic conditions of cows. Adding different fat sources to the diet results in variations in blood NEFA levels and changes in the proportion of FFAs in milk [58]. However, these factors influence the expression of subsequent inflammation-related factors through different receptors, such as FFA and toll-like receptors. In addition, while alpha-tocopherol has the highest concentration and most extended half-life among the vitamin E analogs in mammals, it is only one of the eight members of the vitamin E family. Different vitamin E analogs interact with each other, affecting their metabolism rates [59]. In bovine mammary endothelial cells, both γ-tocopherol and γ-tocotrienol have been shown to reduce the accumulation of ROS and decrease cellular damage [60], illustrating that other vitamin E analogs along with alpha-tocopherol also possess antioxidant functions. In this study, the observed variations in the utilization efficiency of vitamin E among different PBLs and the inconsistent expressions of oxidative stress markers suggest that vitamin E may only partially assist PBLs in overcoming the effects induced by NEFA. Additionally, a report has indicated that providing organic selenium to cows during the transition period can increase glutathione levels in their erythrocytes [61]. Therefore, investigating the combination of different vitamin E analogs or other antioxidants (such as selenium) may contribute to a more comprehensive understanding of the antioxidant capacity and anti-inflammatory effects of the vitamin E family on PBLs under conditions of NEB.

## 5. Conclusions

This study examined whether bovine PBLs improve their performance through vitamin E supplementation under a negative energy balance. It was observed that NEFAs caused an accumulation of lipid oxidation in PBMCs. Pre-treatment with vitamin E reduced the MAD concentration, indicating that vitamin E reduces NEFA-induced oxidation. However, this phenomenon was not observed in PMNs, suggesting that the tolerance and absorption of NEFAs and vitamin E differ between the two peripheral blood leukocytes. In addition, NEFAs increased the expression of specific pro-inflammatory cytokines in both PBLs, whereas vitamin E mitigated the effects caused by NEFAs. Combining the above results, while vitamin E can improve certain functions of peripheral blood leukocytes, its effectiveness varies depending on the cell type. This study confirms that vitamin E has a protective effect against the impact of NEFAs on PBLs. However, some effects cannot be counteracted entirely, such as the increased expression of CSF2 in PMNs. Perhaps using multiple antioxidant strategies could more effectively reduce immune suppression under NEB conditions.

## Figures and Tables

**Table 1:** The qPCR primer sequences.

Gene Symbol	GenBank Accession Number	Forward Primer 5′-3′	Product Size (bp)
		Reverse Primer 3′-5′	
*GAPDH*	NM_001034034.2	CAAGCTCATTTCCTGGTACGAC	130
		AACTCTTCCTCTCGTGCTCC	
*IL-1*β	NM_174093	GACGAGTTTCTGTGTGACGC	149
		ATGCAGAACACCACTTCTCGG	
*IL-6*	NM_173923.2	TGAAAGCAGCAAGGAGACACT	99
		CAAATCGCCTGATTGAACCCAG	
*IL-10*	NM_174088.1	CTGTTGACCCAGTCTCTGCT	216
		GCTCTTGTTTTCGCAGGGC	
*CSF-1*	NM_174026.1	GCCCGTTTTAACTCCGTTCC	180
		TGGCTCTTGATGGCTCCGAC	
*CSF-2*	NM_174027.2	GGCCACCCACTACGAGAAAC	160
		CTGGTTTGGCCTGCTTCACT	
*CSF-3*	NM_174028.1	GCCTGAACCAACTACACGGC	209
		GGCTGAAGTGAAGGTCGGCA	

Abbreviation: GAPDH, glyceraldehyde 3-phosphate dehydrogenase; IL-1β/6/10, interleukin-1β/6/10; CSF-1/2/3, colony-stimulating factors 1/2/3.

**Table 2:** Effect of NEFA and vitamin E pre-treatment combined with NEFA on PBMC and PMN oxidative stress indicators.

Oxidative Stress Indicator	Blank ^1^	NEFA ^2^	VENEFA ^3^
PBMC			
SOD (U/mL)	2.43 ± 0.41	2.35 ± 0.44	2.79 ± 0.46
TBARS (µM/10^7^ cells)	3.01 ± 0.25 ^a^	3.86 ± 0.34 ^b^	3.13 ± 0.24 ^a^
PMN			
SOD (U/mL)	0.4 ± 0.09	0.46 ± 0.1	0.63 ± 0.13
TBARS (µM/10^7^ cells)	3.04 ± 0.3	3.35 ± 0.33	3.37 ± 0.43

^1^ Blank: basal culture medium treatment (4 h) + basal culture medium treatment (8 h).^2^ NEFA: basal culture medium treatment (4 h) +1 mM NEFA treatment (8 h).^3^ VENEFA: 30 μg/mL vitamin E treatment (4 h) + 1 mM NEFA treatment (8 h). Data are presented as means ± standard error. *n* = 12. The different letters indicate significant differences among treatments. (*p* < 0.05). Abbreviation: SOD, superoxide dismutase; TBARS, thiobarbituric acid reactive substance.

**Table 3:** Effect of NEFA and vitamin E pre-treatment combined with NEFA on PBMC and PMN cytokine gene expression.

	PBMC			PMN		
	Blank ^1^	NEFA ^2^	VENEFA ^3^	Blank ^1^	NEFA ^2^	VENEFA ^3^
*IL1β*	1 ± 0.02 ^A^	1.7 ± 0.08 ^C^	1.35 ± 0.17 ^B^	1 ± 0.03	1.49 ± 0.31	1.35 ± 0.27
*IL6*	1 ± 0.03 ^a^	0.86 ± 0.06 ^ab^	0.82 ± 0.11 ^b^	1 ± 0.03	0.99 ± 0.25	1.01 ± 0.34
*IL10*	1 ± 0.03 ^a^	1.37 ± 0.21 ^b^	1.02 ± 0.13 ^a^	1 ± 0.02	2.08 ± 0.96	2.24 ± 1.28
*CSF1*	1 ± 0.03	1.37 ± 0.29	1.09 ± 0.25	1 ± 0.03 ^A^	0.88 ± 0.18 ^AB^	0.61 ± 0.09 ^B^
*CSF2*	1 ± 0.06	1.55 ± 0.4	1.13 ± 0.23	1 ± 0.06 ^A^	1.82 ± 0.28 ^B^	1.42 ± 0.26 ^AB^
*CSF3*	1 ± 0.02 ^a^	4.72 ± 1.78 ^b^	3.07 ± 1.33 ^ab^	1 ± 0.03	1.76 ± 0.59	1.16 ± 0.41

^1^ Blank: basal culture medium treatment (4 h) + basal culture medium treatment (8 h).^2^ NEFA: basal culture medium treatment (4 h) +1 mM NEFA treatment (8 h).^3^ VENEFA: 30 μg/mL vitamin E treatment (4 h) + 1 mM NEFA treatment (8 h). Abbreviation: GAPDH, glyceraldehyde 3-phosphate dehydrogenase; IL-1β/6/10, interleukin-1β/6/10; CSF1/2/3, colony-stimulating factors 1/2/3. Data are presented as mean ± standard error. *n* = 8. Different uppercase letters represent significant levels with *p* < 0.05 between treatments. Different lowercase letters represent significant levels with 0.01 < *p* < 0.05 between treatments.

## Data Availability

The raw data supporting the conclusions of this article will be made available by the authors on request.

## Abbreviations

NEB: negative energy balance; NEFAs: non-esterified fatty acids; ROS: reactive oxygen species; PBLs: peripheral blood leukocytes; PBMCs: peripheral blood mononuclear cells; PMNs: polymorphonuclear neutrophils; MDA: malondialdehyde; NEFA group: basal culture medium treatment (4 h) and 1 mM NEFA treatment (8 h); VENEFA grup: pre-culturing with vitamin E (4 h) before NEFA treatment (8 h).

## References

[1] Herdt T.H. (2000). Ruminant adaptation to negative energy balance. Influences on the etiology of ketosis and fatty liver. Vet. Clin. N. Am. Food Anim. Pract..

[2] Sordillo L.M., Aitken S.L. (2009). Impact of oxidative stress on the health and immune function of dairy cattle. Vet. Immunol. Immunopathol..

[3] Melendez P., Marin M.P., Robles J., Rios C., Duchens M., Archbald L. (2009). Relationship between serum nonesterified fatty acids at calving and the incidence of periparturient diseases in Holstein dairy cows. Theriogenology.

[4] Marutsova V., Binev R., Marutsov P. (2015). Comparative Clinical and Haematological Investigations in Lactating Cows with Subclinical and Clinical Ketosis. Maced. Vet. Rev..

[5] Shimizu T., Morino I., Kitaoka R., Miyamoto A., Kawashima C., Haneda S., Magata F. (2018). Changes of leukocyte counts and expression of pro- and anti-inflammatory cytokines in peripheral leukocytes in periparturient dairy cows with retained fetal membranes. Anim. Sci. J..

[6] Trevisi E., Minuti A. (2018). Assessment of the innate immune response in the periparturient cow. Res. Vet. Sci..

[7] Scalia D., Lacetera N., Bernabucci U., Demeyere K., Duchateau L., Burvenich C. (2006). In vitro effects of nonesterified fatty acids on bovine neutrophils oxidative burst and viability. J. Dairy Sci..

[8] Li C.-Y., Liao Y.-W., Liu C.-S., Cheng C.-Y., Chan J.P.-W., Wang C.-K. (2021). In vitro effects of nonesterified fatty acids and β-hydroxybutyric acid on inflammatory cytokine expression in bovine peripheral blood leukocytes. Ital. J. Anim. Sci..

[9] Uchida K., Beck D.C., Yamamoto T., Berclaz P.Y., Abe S., Staudt M.K., Carey B.C., Filippi M.D., Wert S.E., Denson L.A. (2007). GM-CSF autoantibodies and neutrophil dysfunction in pulmonary alveolar proteinosis. N. Engl. J. Med..

[10] Hidaka T., Akada S., Teranishi A., Morikawa H., Sato S., Yoshida Y., Yajima A., Yaegashi N., Okamura K., Saito S. (2003). Mirimostim (macrophage colony-stimulating factor; M-CSF) improves chemotherapy-induced impaired natural killer cell activity, Th1/Th2 balance, and granulocyte function. Cancer Sci..

[11] Stabel J.R., Kehrli M.E., Thurston J.R., Goff J.P., Boone T.C. (1991). Granulocyte colony-stimulating factor effects on lymphocytes and immunoglobulin concentrations in periparturient cows. J. Dairy Sci..

[12] Kehrli M.E., Goff J.P., Stevens M.G., Boone T.C. (1991). Effects of granulocyte colony-stimulating factor administration to periparturient cows on neutrophils and bacterial shedding. J. Dairy Sci..

[13] McDougall S., LeBlanc S.J., Heiser A. (2017). Effect of prepartum energy balance on neutrophil function following pegbovigrastim treatment in periparturient cows. J. Dairy Sci..

[14] Hamilton J.A. (2008). Colony-stimulating factors in inflammation and autoimmunity. Nat. Rev. Immunol..

[15] Weiss W.P., Todhunter D.A., Hogan J.S., Smith K.L. (1990). Effect of Duration of Supplementation of Selenium and Vitamin E on Periparturient Dairy Cows1. J. Dairy Sci..

[16] Castillo C., Hernández J., Bravo A., Lopez-Alonso M., Pereira V., Benedito J.L. (2005). Oxidative status during late pregnancy in dairy cows. Vet. J..

[17] Politis I., Bizelis I., Tsiaras A., Baldi A. (2004). Effect of vitamin E supplementation on neutrophil function, milk composition and plasmin activity in dairy cows in a commercial herd. J. Dairy Res..

[18] Politis I., Hidiroglou M., Batra T.R., Gilmore J.A., Gorewit R.C., Scherf H. (1995). Effects of vitamin E on immune function of dairy cows. Am. J. Vet. Res..

[19] Sordillo L.M. (2016). Nutritional strategies to optimize dairy cattle immunity1. J. Dairy Sci..

[20] Hall J.A., Bobe G., Vorachek W.R., Kasper K., Traber M.G., Mosher W.D., Pirelli G.J., Gamroth M. (2014). Effect of Supranutritional Organic Selenium Supplementation on Postpartum Blood Micronutrients, Antioxidants, Metabolites, and Inflammation Biomarkers in Selenium-Replete Dairy Cows. Biol. Trace Elem. Res..

[21] Bouwstra R.J., Goselink R.M.A., Dobbelaar P., Nielen M., Newbold J.R., van Werven T. (2008). The Relationship Between Oxidative Damage and Vitamin E Concentration in Blood, Milk, and Liver Tissue from Vitamin E Supplemented and Nonsupplemented Periparturient Heifers. J. Dairy Sci..

[22] Romana Kadek J.F., Mikulková K., Illek J. (2022). Concentration of vitamin E in bovine plasma and erythrocytes. Acta Vet. Brno..

[23] National Research Council, Committee on Animal Nutrition, Subcommittee on Dairy Cattle Nutrition (2001). Nutrient Requirements of Dairy Cattle.

[24] Horn P., Bork S., Wagner W., Vemuri M., Chase L.G., Rao M.S. (2011). Standardized Isolation of Human Mesenchymal Stromal Cells with Red Blood Cell Lysis. Mesenchymal Stem Cell Assays and Applications.

[25] Douglas G.N., Rehage J., Beaulieu A.D., Bahaa A.O., Drackley J.K. (2007). Prepartum Nutrition Alters Fatty Acid Composition in Plasma, Adipose Tissue, and Liver Lipids of Periparturient Dairy Cows. J. Dairy Sci..

[26] Lacetera N., Scalia D., Franci O., Bernabucci U., Ronchi B., Nardone A. (2004). Short Communication: Effects of Nonesterified Fatty Acids on Lymphocyte Function in Dairy Heifers. J. Dairy Sci..

[27] Adolfsson O., Huber B.T., Meydani S.N. (2001). Vitamin E-Enhanced IL-2 Production in Old Mice: Naive But Not Memory T Cells Show Increased Cell Division Cycling and IL-2-Producing Capacity1. J. Immunol..

[28] Wankhade P.R., Manimaran A., Kumaresan A., Jeyakumar S., Ramesha K.P., Sejian V., Rajendran D., Varghese M.R. (2017). Metabolic and immunological changes in transition dairy cows: A review. Vet. World.

[29] Yang W., Zhang B., Xu C., Zhang H., Cheng X. (2019). Effects of Ketosis in Dairy Cows on Blood Biochemical Parameters, Milk Yield and Composition, and Digestive Capacity. J. Vet. Res..

[30] Robinson T.L., Sutherland I.A., Sutherland J. (2007). Validation of candidate bovine reference genes for use with real-time PCR. Vet. Immunol. Immunopathol..

[31] Hayirli A., Grummer R.R., Nordheim E.V., Crump P.M. (2003). Models for Predicting Dry Matter Intake of Holsteins During the Prefresh Transition Period. J. Dairy Sci..

[32] Weber C., Hametner C., Tuchscherer A., Losand B., Kanitz E., Otten W., Singh S.P., Bruckmaier R.M., Becker F., Kanitz W. (2013). Variation in fat mobilization during early lactation differently affects feed intake, body condition, and lipid and glucose metabolism in high-yielding dairy cows. J. Dairy Sci..

[33] van der Drift S.G.A., Houweling M., Schonewille J.T., Tielens A.G.M., Jorritsma R. (2012). Protein and fat mobilization and associations with serum β-hydroxybutyrate concentrations in dairy cows. J. Dairy Sci..

[34] Reynolds C.K., Aikman P.C., Lupoli B., Humphries D.J., Beever D.E. (2003). Splanchnic Metabolism of Dairy Cows During the Transition From Late Gestation Through Early Lactation. J. Dairy Sci..

[35] Pullen D.L., Liesman J.S., Emery R.S. (1990). A species comparison of liver slice synthesis and secretion of triacylglycerol from nonesterified fatty acids in media2. J. Anim. Sci..

[36] Herdt T.H., Wensing T., Haagsman H.P., van Golde L.M.G., Breukink H.J. (1988). Hepatic Triacylglycerol Synthesis during a Period of Fatty Liver Development in Sheep2. J. Anim. Sci..

[37] Shu C. (2014). Investigation on the Relationship of Insulin Resistance and Ketosis in Dairy Cows. J. Vet. Sci. Technol..

[38] Li Y., Ding H.Y., Wang X.C., Feng S.B., Li X.B., Wang Z., Liu G.W., Li X.W. (2016). An association between the level of oxidative stress and the concentrations of NEFA and BHBA in the plasma of ketotic dairy cows. J. Anim. Physiol. Anim. Nutr..

[39] Shi X., Li D., Deng Q., Li Y., Sun G., Yuan X., Song Y., Wang Z., Li X., Li X. (2015). NEFAs activate the oxidative stress-mediated NF-κB signaling pathway to induce inflammatory response in calf hepatocytes. J. Steroid Biochem. Mol. Biol..

[40] Li C., Huang J., Chen X., Yan Y., Li L., Zhao W. (2022). Transcriptome Analysis Reveals That NEFA and β-Hydroxybutyrate Induce Oxidative Stress and Inflammatory Response in Bovine Mammary Epithelial Cells. Metabolites.

[41] Sharma N., Singh N.K., Singh O.P., Pandey V., Verma P.K. (2011). Oxidative Stress and Antioxidant Status during Transition Period in Dairy Cows. Asian-Australas. J. Anim. Sci..

[42] Wagner B.A., Buettner G.R., Burns C.P. (1994). Free radical-mediated lipid peroxidation in cells: Oxidizability is a function of cell lipid bis-allylic hydrogen content. Biochemistry.

[43] Trommer S., Leimert A., Bucher M., Schumann J. (2018). Polyunsaturated Fatty Acids Induce ROS Synthesis in Microvascular Endothelial Cells. Adv. Exp. Med. Biol..

[44] Burns C., Welshman I., Spector A. (1976). Differences in free fatty acid and glucose metabolism of human blood neutrophils and lymphocytes. Blood.

[45] Politis I., Hidiroglou N., White J.H., Gilmore J.A., Williams S.N., Scherf H., Frigg M. (1996). Effects of vitamin E on mammary and blood leukocyte function, with emphasis on chemotaxis, in periparturient dairy cows. Am. J. Vet. Res..

[46] Dang A.K., Prasad S., De K., Pal S., Mukherjee J., Sandeep I.V.R., Mutoni G., Pathan M.M., Jamwal M., Kapila S. (2013). Effect of supplementation of vitamin E, copper and zinc on the in vitro phagocytic activity and lymphocyte proliferation index of peripartum Sahiwal (*Bos indicus*) cows. J. Anim. Physiol. Anim. Nutr..

[47] Schäfers S., von Soosten D., Meyer U., Drong C., Frahm J., Tröscher A., Pelletier W., Sauerwein H., Dänicke S. (2018). Influence of conjugated linoleic acids and vitamin E on biochemical, hematological, and immunological variables of dairy cows during the transition period. J. Dairy Sci..

[48] Khatti A., Mehrotra S., Patel P.K., Singh G., Maurya V.P., Mahla A.S., Chaudhari R.K., Das G.K., Singh M., Sarkar M. (2017). Supplementation of vitamin E, selenium and increased energy allowance mitigates the transition stress and improves postpartum reproductive performance in the crossbred cow. Theriogenology.

[49] Ndiweni N., Finch J.M. (1995). Effects of in vitro supplementation of bovine mammary gland macrophages and peripheral blood lymphocytes with α-tocopherol and sodium selenite: Implications for udder defences. Vet. Immunol. Immunopathol..

[50] Weiss W.P., Hogan J.S., Wyatt D.J. (2009). Relative bioavailability of all-rac and RRR vitamin E based on neutrophil function and total α-tocopherol and isomer concentrations in periparturient dairy cows and their calves. J. Dairy Sci..

[51] Palladini G., Di Pasqua L.G., Berardo C., Siciliano V., Richelmi P., Mannucci B., Croce A.C., Rizzo V., Perlini S., Vairetti M. (2019). Fatty Acid Desaturase Involvement in Non-Alcoholic Fatty Liver Disease Rat Models: Oxidative Stress Versus Metalloproteinases. Nutrients.

[52] Grajchen E., Loix M., Baeten P., Côrte-Real B.F., Hamad I., Vanherle S., Haidar M., Dehairs J., Broos J.Y., Ntambi J.M. (2023). Fatty acid desaturation by stearoyl-CoA desaturase-1 controls regulatory T cell differentiation and autoimmunity. Cell. Mol. Immunol..

[53] Li P., Li L., Zhang C., Cheng X., Zhang Y., Guo Y., Long M., Yang S., He J. (2019). Palmitic Acid and β-Hydroxybutyrate Induce Inflammatory Responses in Bovine Endometrial Cells by Activating Oxidative Stress-Mediated NF-κB Signaling. Molecules.

[54] Bahramian N., Östergren-Lundén G., Bondjers G., Olsson U. (2004). Fatty acids induce increased granulocyte macrophage-colony stimulating factor secretion through protein kinase C-activation in THP-1 macrophages. Lipids.

[55] O’Mahony D.S., Pham U., Iyer R., Hawn T.R., Liles W.C. (2008). Differential constitutive and cytokine-modulated expression of human Toll-like receptors in primary neutrophils, monocytes, and macrophages. Int. J. Med. Sci..

[56] Sattler M., Winkler T., Verma S., Byrne C.H., Shrikhande G., Salgia R., Griffin J.D. (1999). Hematopoietic Growth Factors Signal Through the Formation of Reactive Oxygen Species. Blood.

[57] Cárcamo J.M., Bórquez-Ojeda O., Golde D.W. (2002). Vitamin C inhibits granulocyte macrophage–colony-stimulating factor–induced signaling pathways. Blood.

[58] Dirandeh E., Towhidi A., Ansari Z., Zeinoaldini S., Ganjkhanlou M. (2016). Effects of Dietary Supplementation with Different Polyunsaturated Fatty Acids on Expression of Genes Related to Somatotropic Axis Function in the Liver, Selected Blood Indicators, Milk Yield and Milk Fatty Acids Profile in Dairy Cows. Ann. Anim. Sci..

[59] Sontag T.J., Parker R.S. (2007). Influence of major structural features of tocopherols and tocotrienols on their ω-oxidation by tocopherol-ω-hydroxylase. J. Lipid Res..

[60] Kuhn M.J., Sordillo L.M. (2021). Vitamin E analogs limit in vitro oxidant damage to bovine mammary endothelial cells. J. Dairy Sci..

[61] Gong J., Xiao M. (2018). Effect of Organic Selenium Supplementation on Selenium Status, Oxidative Stress, and Antioxidant Status in Selenium-Adequate Dairy Cows During the Periparturient Period. Biol. Trace Elem. Res..

